# Advanced Carbon Nanostructures: Synthesis, Properties, and Applications

**DOI:** 10.3390/nano13071268

**Published:** 2023-04-03

**Authors:** Marianna V. Kharlamova, Christian Kramberger, Alexander I. Chernov

**Affiliations:** 1 Centre for Advanced Materials Application (CEMEA), Slovak Academy of Sciences, Dúbravská cesta 5807/9, 845 11 Bratislava, Slovakia; 2 Phystech School of Biological, and Medical Physics, Moscow Institute of Physics and Technology (National Research University), 9 Institutskiy Per., Dolgoprudny 141701, Russia; 3 Institute of Materials Chemistry, Vienna University of Technology, Getreidemarkt 9/BC/2, 1060 Vienna, Austria; 4 Faculty of Physics, University of Vienna, Strudlhofgasse 4, 1090 Vienna, Austria; christian.kramberger-kaplan@univie.ac.at; 5 Russian Quantum Center, Skolkovo Innovation City, 30 Bolshoy Bulvar, Moscow 121205, Russia; ach@rqc.ru; 6 Center for Photonics and 2D Materials, Moscow Institute of Physics and Technology (National Research University), 9 Institutskiy Per., Dolgoprudny 141701, Russia

Carbon nanomaterials are a class of materials that include allotropic modifications of carbon [1]. These include 0D allotropes—carbon dots, 1D—carbon nanotubes (CNT), 2D—graphene, and 3D—graphite. Investigating the chemistry of carbon allotropes has led to many new, interesting discoveries in all the above groups [2]. The physics of carbon allotropes is an interesting topic which has attracted the attention of researchers [3]. The scope of our Special Issue, “Advanced Carbon Nanostructures: Synthesis, Properties, and Applications”, is advanced carbon nanostructures, such as carbon nanotubes, graphene, graphene nanoribbons, and 2D van der Walls heterostructures. The papers presented with discuss aspects of synthesis, sorting, functionalization, and characterization of chemical and physical properties, leading to many interesting applications being highlighted and experimental, theoretical, and modelling aspects being discussed. In the Special Issue, 15 papers were published, consisting of 2 reviews [4,5] and 13 research papers [6,7,8,9,10,11,12,13,14,15,16,17,18].

In this Editorial, we summarize the published papers.

In a review paper [4] invited by the Guest Editors, the authors present a review of metal and metal-halogenide-filled single-walled carbon nanotubes (SWCNTs). The kinetic and electronical properties of the filled SWCNTs and their applications are considered, and the results of a quantitative analysis of charge transfer are discussed. A comparison of the electronic properties of SWCNTs filled with different substances is performed (Figure 1).

In [5], the peculiarities of machine learning for property prediction of materials are discussed (Figure 2). The authors summarize the significant contribution of machine learning to property prediction in two fields: material property detection and degradation detection. The outstanding challenges of description and perspectives are presented, including method and application innovation, principle exploration, and data support.

In [6], the authors synthesized carbon hybrid materials mainly using the gas-phase pyrolysis method. They performed characterization of hybrids by microscopy and thermogravimetric analysis (Figure 3). This allowed them to study the morphology and composition of the carbon nanotubes. The authors also discussed the examples of potential applications of carbon hybrid materials.

In [7], the authors performed photoemission spectroscopy studies of graphene. The contribution of epoxide and hydroxyl groups in C 1s X-ray photoelectron spectra were experimentally revealed. In the valence band spectra, the molecular orbitals of these functionalities were determined. The density functional theory calculations corresponded to the experimental findings (Figure 4).

In [8], the authors prepared CNT/Mg composites using a procedure including grinding manganese powder with distributing CNTs. The concentration of Ni-coated CNTs defined the uniform distribution. The composites were analyzed by transmission electron microscopy (TEM), scanning electron microscopy (SEM), X-ray diffraction, and compression tests (Figure 5).

In [9], hierarchical CNTs/AZ61 composites were synthesized by dispersing Ni-coated CNTs in AZ61 matrix by ball milling. It was shown that the fracture strain and compressive strength were improved as compared with homogeneous CNTs/AZ61. With an increase in CNT concentration, the fracture strain was gradually lowered because of the agglomeration of CNTs, whereas the compressive strength did not change (Figure 6).

In [10], the authors investigated the impact of sp^2^ concentration in nanodiamonds catalysts for acetylene hydrochlorination. They were treated at 500 °C, 700 °C, 900 °C, and 1100 °C, which led to increase in the sp^2^ concentration (Figure 7), whereas the catalytic activity showed nonmonotonic behavior. The highest catalytic activity was observed for nanodiamonds calcinated at 900 °C with a sp^2^ concentration of 43.9%.

In [11], CNTs/refined-AZ61 composites were synthesized by dispersing Cu-coated CNTs in an AZ61 matrix by ball milling and hot-pressing sintering. It was shown that at a concentration less than or equal to 1 vol.%, CNTs are uniformly distributed, and the yield strength and compressive strength of composites improves with increasing CNT concentrations (Figure 8).

In [12], the authors investigated the morphology of annealed SWCNTs (Figure 9). The SWCNTs were filled with europium (III) chloride and then were investigated by SEM, TEM, and energy-dispersive X-ray analysis, confirming the filling and closed ends. It was shown that the apparent surface area of SWCNTs, amount of closed ends, and content of filler can be tuned by annealing.

In [13], the authors investigated temperature-dependent inner SWCNT growth in nickelocene-, cobaltocene-, and ferrocene-filled SWCNTs with Raman spectroscopy. The influence of temperature, diameter, and metal catalyst type on the growth of inner SWCNTs was revealed (Figure 10). The growth of 36 chiralities of inner SWCNTs was investigated. It was shown that smaller-diameter tubes grow faster for all three catalysts.

In [14], the authors suggested the original method of calculation of piezoelectric coefficient of CNTs. They investigated the dependence of piezoelectric coefficient of CNTs on temperature of growth and thickness of catalyst layer. They found a correlation between the effective piezoelectric coefficient of CNTs, their defectiveness, and diameter (Figure 11).

In [15], the authors synthesized a hierarchical porous carbon material (HPC) using sisal fiber (SF) as a precursor. H_3_PW_12_O_40_·24H_2_O (HPW) was dispersed on the surface of SF-HPC. HPW/SF-HPCs were characterized by a high surface area. Their X-ray diffraction analysis (XRD) and FT-IR spectra are shown in Figure 12. HPW/SF-HPW had a superb catalytic activity for oxidative desulfurization.

In [16], the growth kinetics of inner SWCNTs were investigated for nickelocene- and cobaltocene-filled SWCNTs. Nine individual-chirality inner tubes, (8,8), (12,3), (13,1), (9,6), (10,4), (11,2), (11,1), (9,3), and (9,2), were investigated. It was shown that the growth rates were larger for smaller-diameter SWCNTs. The activation energies showed dependence on the diameter of SWCNTs, which is shown in Figure 13.

In [17], metallic nickelocene-filled SWCNTs were obtained using density-gradient ultracentrifugation of filled carbon nanotubes. The electronic properties of metallic nickelocene-filled SWCNTs were investigated with Raman spectroscopy and X-ray photoelectron spectroscopy (XPS). It was shown that the electronic properties of sorted filled SWCNTs can be tuned by annealing at 360–1200 °C (Figure 14).

In [18], the authors synthesized nanohybrids consisting of oxidized single-walled carbon nanohorns (ox-SWCNH)-SnO_2_-polyvinylpyrrolidone (PVP) with stoichiometry 1/1/1 and 2/1/1, and ox-SWCNH-ZnO-PVP with stoichiometry 5/2/1 and 5/3/2 (mass ratios). Raman spectra of ox-SWCNH-SnO_2_-PVP are presented in Figure 15. The nanohybrids were tested as sensing films for ethanol vapor detection in dry air.

We acknowledge all of the authors for their excellent contributions and invite you to collaborate further by sending your best works to the second edition of our Special Issue: “Advanced Carbon Nanostructures: Synthesis, Properties, and Applications II”.

## Figures and Tables

**Figure 1:**
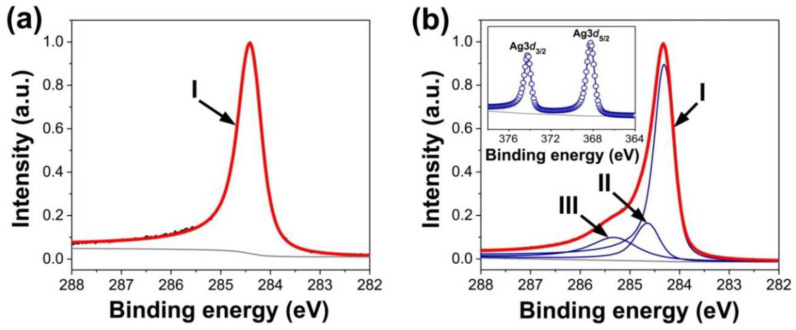
The C 1s and Ag 3D X-ray photoelectron spectroscopy data of the pristine (**a**) and silver-filled SWCNTs (**b**). The roman numbers denote the components of the spectra. M. V. Kharlamova et al. Donor doping of single-walled carbon nanotubes by filling of channels with silver, *Journal of Experimental and Theoretical Physics*, V. 115, № 3, pp. 485–491, 2012, reproduced with permission from SNCSC [19].

**Figure 2:**
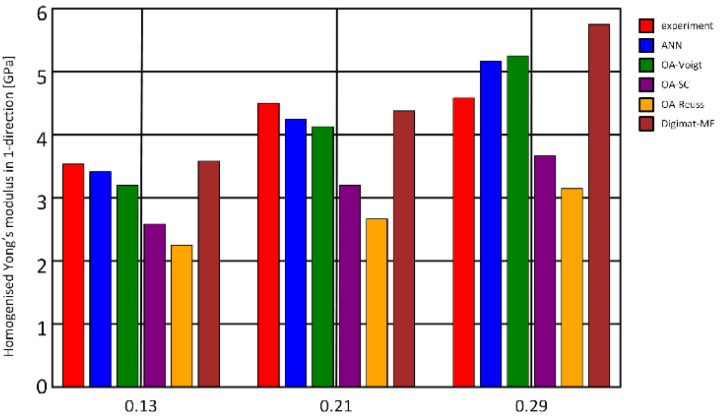
Machine learning for property prediction of materials. ANN—artificial neural networks. OA—orientation averaging. Digimat-MF—mean-field predictions. Reprinted from Ref. [20]. Copyright 2021 The Authors. Published by Elsevier Ltd. This is an open access article under the CC BY license.

**Figure 3:**
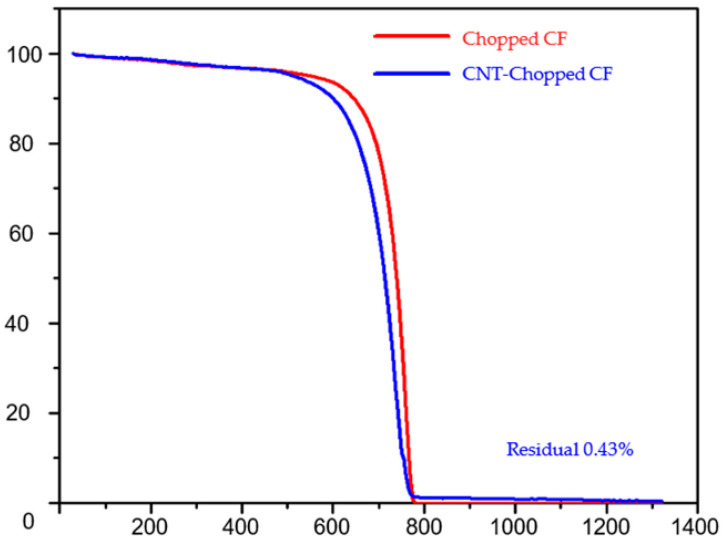
Thermogravimetric analysis data for chopped carbon fiber (CF) and CNT-integrated chopped fiber. Reprinted from Ref. [6]. Copyright 2023 by the authors. Licensee MDPI, Basel, Switzerland. This article is an open access article distributed under the terms and conditions of the Creative Commons Attribution (CC BY) license.

**Figure 4:**
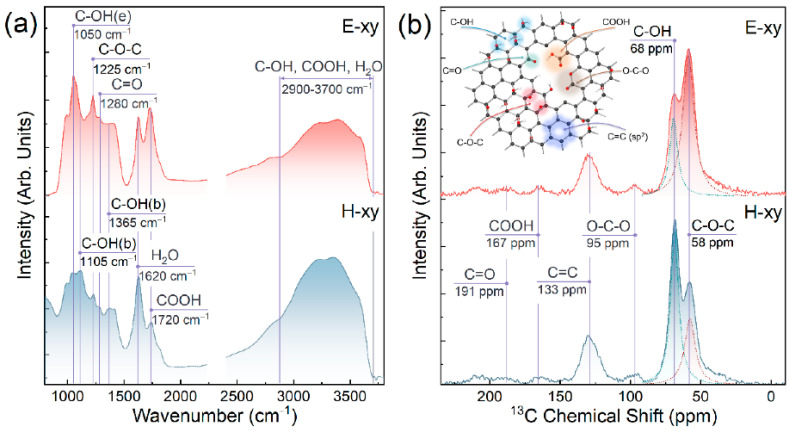
(**a**) Fourier–transformed infrared (FT–IR) spectra and (**b**) solid–state ^13^C nuclear magnetic resonance (NMR) spectroscopy of E–xy and H–xy graphenes, and schematics of graphene chemistry. Reprinted from Ref. [7]. Copyright 2022 by the authors. Licensee MDPI, Basel, Switzerland. This article is an open access article distributed under the terms and conditions of the Creative Commons Attribution (CC BY) license.

**Figure 5:**
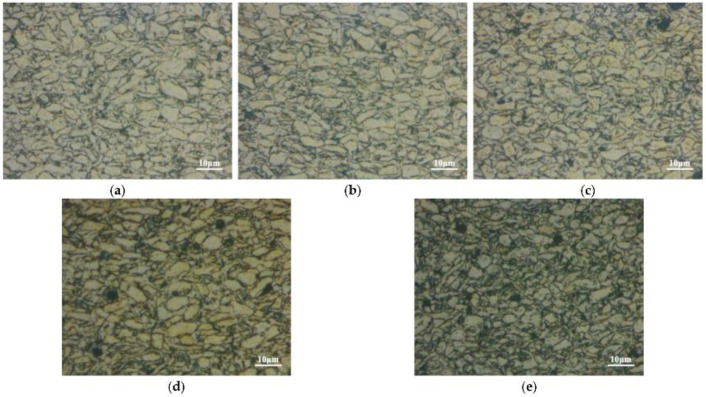
Images of CNT/Mg composites: (**a**) 0.5 vol.% CNTs; (**b**) 1 vol.% CNTs; (**c**) 1.5 vol.% CNTs; (**d**) 2 vol.% CNTs; (**e**) 2.5 vol.% CNTs. Reprinted from Ref. [8]. Copyright 2022 by the authors. Licensee MDPI, Basel, Switzerland. This article is an open access article distributed under the terms and conditions of the Creative Commons Attribution (CC BY) license.

**Figure 6:**
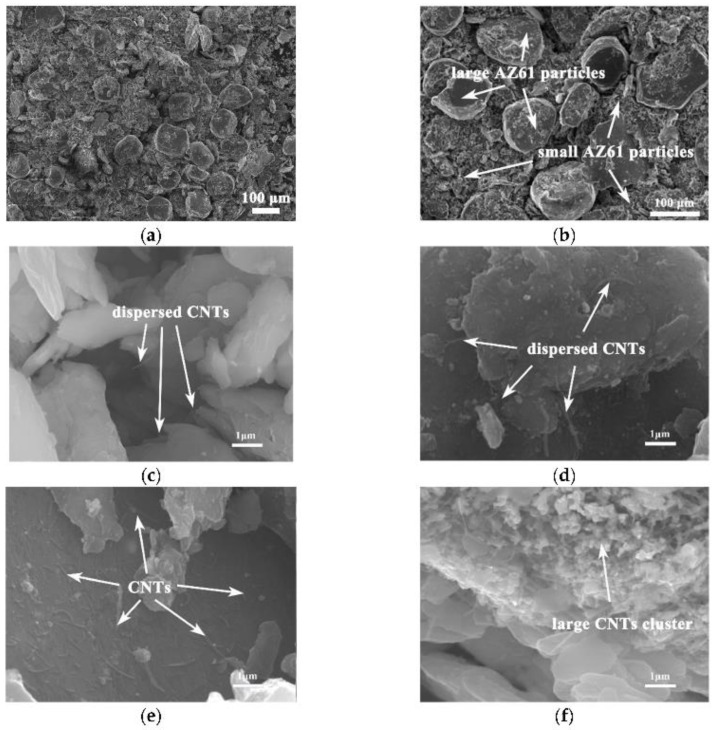
The SEM images of CNTs/AZ61 composites: (**a**,**b**) low-magnification images, (**c**) 0.5% CNTs, (**d**) 0.75% CNTs, (**e**) 1% CNTs, and (**f**) 1.25% CNTs. Reprinted from Ref. [9]. Copyright 2022 by the authors. Licensee MDPI, Basel, Switzerland. This article is an open access article distributed under the terms and conditions of the Creative Commons Attribution (CC BY) license.

**Figure 7:**
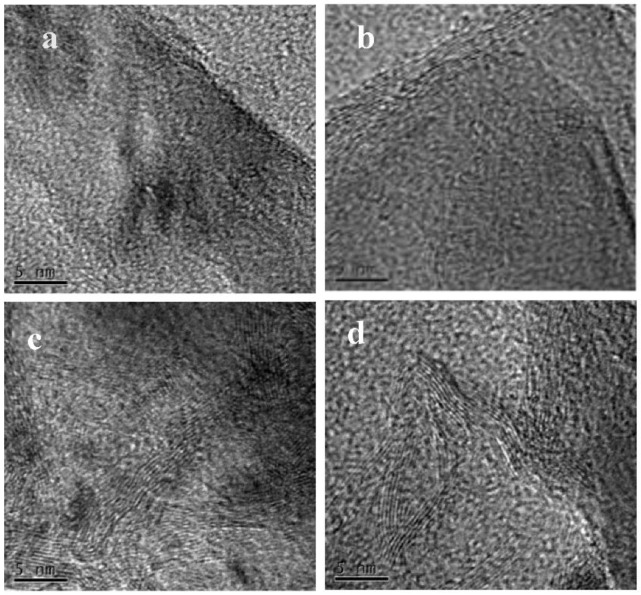
The TEM images of nanodiamond catalysts calcinated at 500 °C (**a**), 700 °C (**b**), 900 °C (**c**), and 1100 °C (**d**). Reprinted from Ref. [10]. Copyright 2022 by the authors. Licensee MDPI, Basel, Switzerland. This article is an open access article distributed under the terms and conditions of the Creative Commons Attribution (CC BY) license.

**Figure 8:**
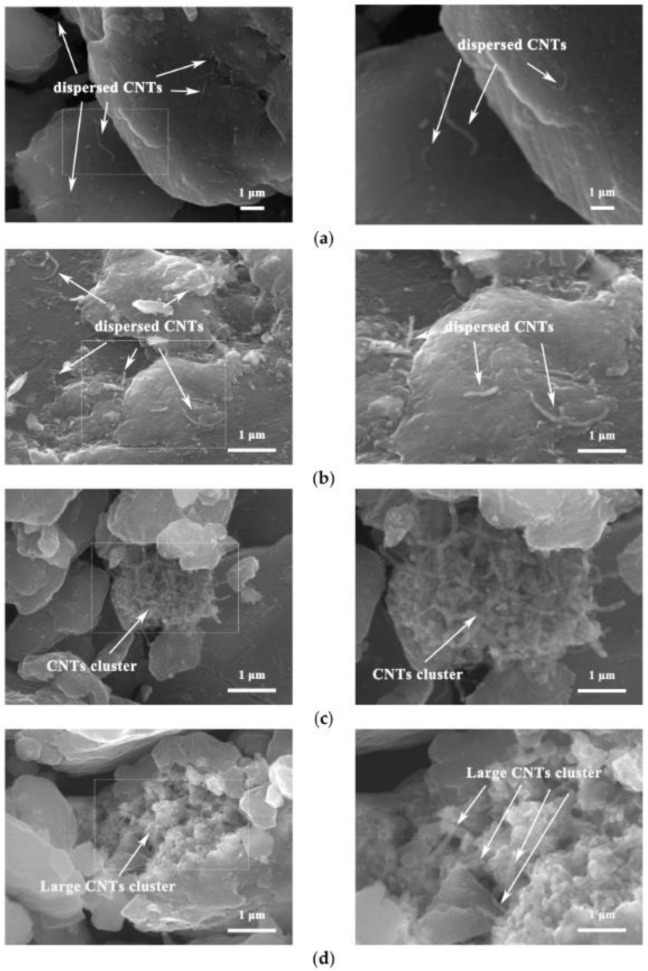
The SEM images of CNTs/refined-AZ61 composites: (**a**) 0.5% CNTs, (**b**) 1% CNTs, (**c**) 1.5% CNTs, (**d**) 2% CNTs. Reprinted from Ref. [11]. Copyright 2022 by the authors. Licensee MDPI, Basel, Switzerland. This article is an open access article distributed under the terms and conditions of the Creative Commons Attribution (CC BY) license.

**Figure 9:**
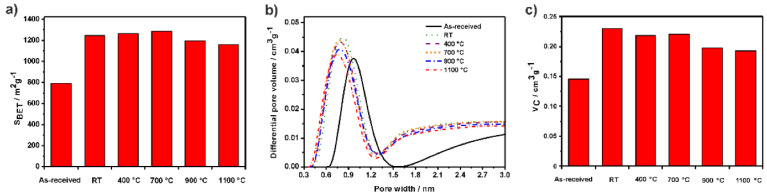
(**a**) S_BET_, the apparent specific surface area. (**b**) Nonlocal density functional theory (NLDFT) pore size distribution. (**c**) V_C_, cumulative volume for as–received, purified (RT) and annealed SWCNTs. Reprinted from Ref. [12]. Copyright 2021 by the authors. Licensee MDPI, Basel, Switzerland. This article is an open access article distributed under the terms and conditions of the Creative Commons Attribution (CC BY) license.

**Figure 10:**
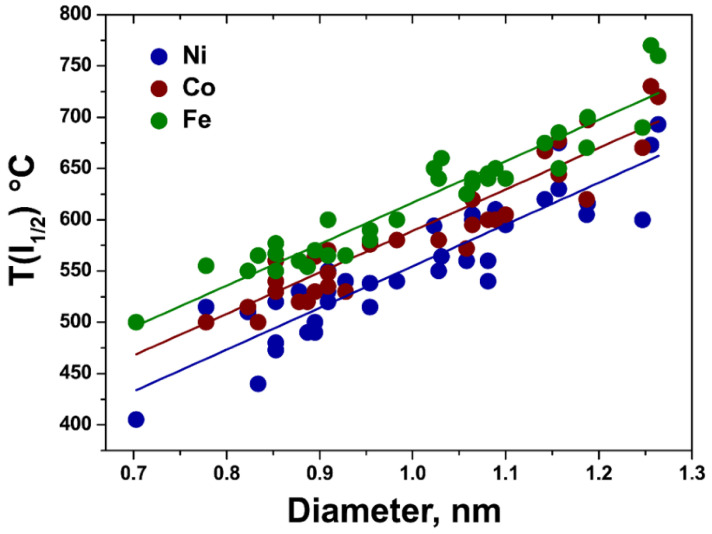
The dependence of growth temperature T(I_1/2_) on diameter for metallocene-grown inner carbon nanotubes. The linear fits are denoted as solid lines. Reprinted from Ref. [13]. Copyright 2021 by the authors. Licensee MDPI, Basel, Switzerland. This article is an open access article distributed under the terms and conditions of the Creative Commons Attribution (CC BY) license.

**Figure 11:**
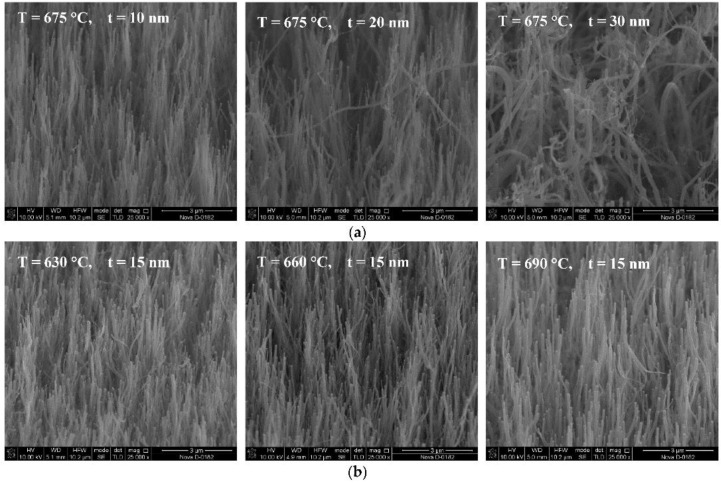
(**a**) The SEM images of CNT arrays grown at 675 °C and different catalyst layer thicknesses *t* = 10–30 nm, and (**b**) at catalyst layer thickness *t* = 15 nm, and at different temperatures 630–690 °C. Reprinted from Ref. [14]. Copyright 2021 by the authors. Licensee MDPI, Basel, Switzerland. This article is an open access article distributed under the terms and conditions of the Creative Commons Attribution (CC BY) license.

**Figure 12:**
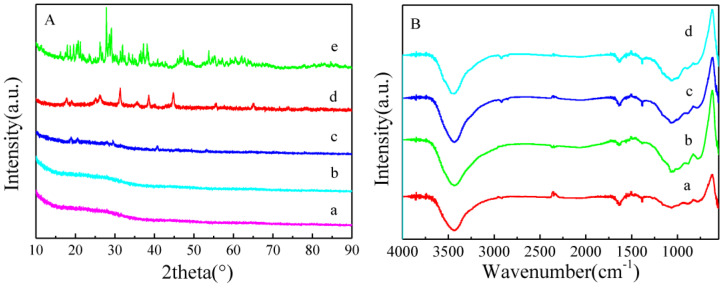
XRD (**A**) and FT-IR spectra (**B**) of (a) SF-HPC, (b) 1% HPW/SF-HPC, (c) 5% HPW/SF-HPC, (d) 10% HPW/SF-HPC, and (e) HPW. Reprinted from Ref. [15]. Copyright 2021 by the authors. Licensee MDPI, Basel, Switzerland. This article is an open access article distributed under the terms and conditions of the Creative Commons Attribution (CC BY) license.

**Figure 13:**
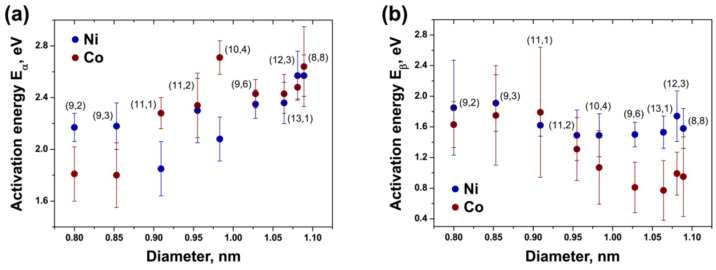
The dependence of activation energy E_α_ (**a**) and E_β_ (**b**) of growth of inner SWCNTs on diameter for nickelocene- and cobaltocene-filled SWCNTs. The chiralities of inner tubes are labelled. Reprinted from Ref. [16]. Copyright 2021 by the authors. Licensee MDPI, Basel, Switzerland. This article is an open access article distributed under the terms and conditions of the Creative Commons Attribution (CC BY) license.

**Figure 14:**
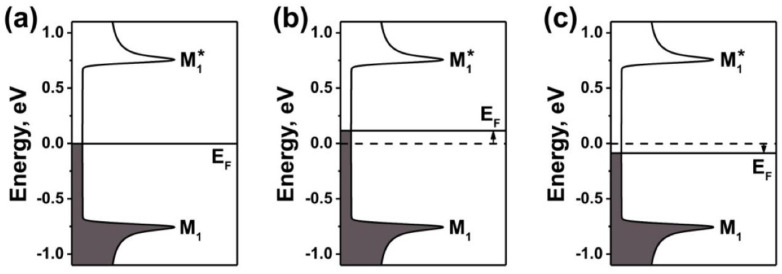
The schematics of the band structures of metallic SWCNTs (E_F_ is the Fermi level and M_1_, and M_1_* are van Hove singularities) (**a**), upshift (**b**), and downshift (**c**) of the Fermi level of treated SWCNTs. Reprinted from Ref. [17]. Copyright 2021 by the authors. Licensee MDPI, Basel, Switzerland. This article is an open access article distributed under the terms and conditions of the Creative Commons Attribution (CC BY) license.

**Figure 15:**
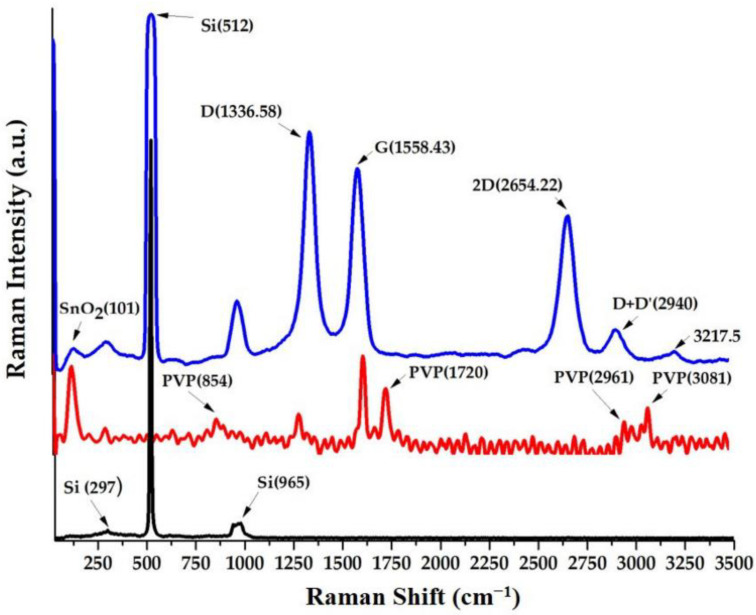
Raman spectra of ox-SWCNH-SnO_2_-PVP with stoichiometry 1/1/1. Reprinted from Ref. [18]. Copyright 2020 by the authors. Licensee MDPI, Basel, Switzerland. This article is an open access article distributed under the terms and conditions of the Creative Commons Attribution (CC BY) license.
